# Supplementary material for: Local infiltration analgesia with bupivacaine and adrenaline does not reduce perioperative blood loss in total hip arthroplasty
Source: PLoS One. 2021 Sep 9;16(9):e0257202. doi: 10.1371/journal.pone.0257202 (PMC8428560; doi:10.1371/journal.pone.0257202)
Supplement: S2 File — (DOCX) [file pone.0257202.s002.docx]

**Supplementary tables for patients’ evaluation in age groups.**

**Suppl. Tab. 1a**

**Patient characteristics (age 60-69 years old)**

|  | Infiltrated group  (*n* = 13) | Not infiltrated group  (*n* = 12) | Statistics |
| --- | --- | --- | --- |
| Weight (kilograms) | 80.77  SD = 15.7  R: 55-108 | 79.58  SD = 13.1  R: 62-100 | p = 0.93 |
| BMI | 26.95  SD = 15.7  R: 21 - 32 | 26.47  SD = 2.36  R: 22 - 29 | p = 0.4 |
| Acetabulum size*^a^* (mm) | 56.18  SD = 3.76  R: 50-60 | 57.4  SD = 3.1  R: 52-62 | p = 0.06 |
| Stem size*^a^* | 12.86  SD = 2.1  R: 10-18 | 12.85  SD = 3.25  R: 7.5 – 17.5 | p = 0.88 |
| Operative time in min. | 86.92  SD = 12  R: 60-105 | 96.66  SD = 13.28  R: 70-115 | p = 0.09 |

*^a^* The sample sizes for the acetabulum and the stem were *n* = 11 patients in the infiltrated group and *n* = 10 patients in the not infiltrated group

**Suppl. Tab. 1b**

**Patient characteristics (age 70-79 years old)**

|  | Infiltrated group  (*n* = 27) | Not infiltrated group  (*n* = 22) | Statistics |
| --- | --- | --- | --- |
| Weight (kilograms) | 75.33  SD = 11.36  R: 56-110 | 74.22  SD = 11.45  R: 55-102 | p = 0.74 (t-Stud) |
| BMI | 25.85  SD = 2.8  R: 21 - 32 | 25.04  SD = 2.39  R: 21 - 32 | p = 0.49 |
| Acetabulum size*^a^* (mm) | 54.66  SD = 3.26  R: 50-62 | 54.38  SD = 5.11  R: 44-65 | p = 0.001 |
| Stem size*^a^* | 11.86  SD = 3.26  R: 7.5-17.5 | 11.36  SD = 2.35  R: 7.5 – 17.5 | p = 0.97 |
| Operative time in min. | 90.92  SD = 17  R: 55-125 | 88.63  SD = 12.8  R: 60-110 | p = 0.61 (t-Stud) |

*^a^* The sample sizes for the acetabulum and the stem were *n* = 18 patients in the infiltrated group and *n* = 18 patients in the not infiltrated group

**Suppl. Tab. 2a**

**The comparison of preoperative morphology and INR results (age 60-69 years old).**

|  | Infiltrated group  (*n* = 13) | Not infiltrated group  (*n* = 12) | Statistics |
| --- | --- | --- | --- |
| Hb (g/dl) | 14.92  SD = 1.21  R: 12.5 – 16.5 | 13.78  SD = 1.2  R: 11.5 – 15,9 | p = 0.58 |
| HTC (%) | 44  SD = 3.44  R: 38 – 49.1 | 41  SD = 2.7  R: 36 - 45 | p = 0.98 |
| RBC (T/l) | 4.92  SD = 0.36  R: 4.21 – 5.45 | 4.5  SD = 0.25  R: 4.11 – 4.88 | p = 0.37 |
| PLT (G/l) | 243  SD = 64  R: 160 - 384 | 263  SD = 85  R: 178 - 425 | p = 0.89 |
| INR | 1.01  SD = 0.1  R: 0.85 – 1.19 | 0.98  SD = 0.09  R: 0.84 – 1.11 | p = 0.73 |

**Suppl. Tab. 2b**

**The comparison of preoperative morphology and INR results (age 70-79 years old).**

|  | Infiltrated group  (*n* = 27) | Not infiltrated group  (*n* = 22) | Statistics |
| --- | --- | --- | --- |
| Hb (g/dl) | 13.55  SD = 0.96  R: 11.7 – 15.1 | 13.98  SD = 1.2  R: 12 – 16.4 | p = 0.78 |
| HTC (%) | 40.6  SD = 2.56  R: 35.4 – 45.2 | 41  SD = 2.8  R: 37- 46 | p = 0.43 |
| RBC (T/l) | 4.61  SD = 0.33  R: 4.06 – 5.25 | 4.74  SD = 0.41  R: 4.02 – 5.44 | p = 0.72 |
| PLT (G/l) | 266  SD = 89  R: 153 - 456 | 229  SD = 65  R: 140 - 378 | p = 0.23 (t-Stud) |
| INR | 1.04  SD = 0.09  R: 0.85 – 1.2 | 1.06  SD = 0.1  R: 0.92 – 1.32 | p = 0.88 |

**Suppl. Tab. 3a**

**The comparison of postoperative hemoglobin levels (Hb) and hematocrit (HTC) (age 60-69 years old).**

| Postoperative day |  | Infiltrated group  (*n* = 13) | Not infiltrated group  (*n* = 12) | Statistics |
| --- | --- | --- | --- | --- |
| Day 1 | Hb (g/dl) | 11.39  SD = 1.01  R: 9.2 – 13.5 | 11.09  SD = 1.13  R: 9 – 13.2 | p = 0.78 (t-Stud) |
|  | HTC (%) | 33.6  SD = 3.19  R: 27.2 – 39.5 | 32  SD = 3.5  R: 23 - 38 | p = 0.93 |
| Day 4 | Hb (g/dl) | 10.29  SD = 1.31  R: 7.2 – 12.3 | 10.44  SD = 1.13  R: 9 – 13.2 | p = 0.54 (t-Stud) |
|  | HTC (%) | 30.4  SD = 3.95  R: 20.4 –35.7 | 31  SD = 2.9  R: 27 - 39 | p = 0.67 |
| Discharge | Hb (g/dl) | 10.82  SD = 0.6  R: 10.1 – 12.3 | 10.58  SD = 1  R: 9.5 – 13.2 | p = 0.86 |
|  | HTC (%) | 32  SD = 1.72  R: 29.3 – 35.7 | 31  SD = 2.6  R: 29 - 39 | p = 0.54 |

**Suppl. Tab. 3b**

**The comparison of postoperative hemoglobin levels (Hb) and hematocrit (HTC) (age 70-79 years old).**

| Postoperative day |  | Infiltrated group  (*n* = 27) | Not infiltrated group  (*n* = 22) | Statistics |
| --- | --- | --- | --- | --- |
| Day 1 | Hb (g/dl) | 11.06  SD = 1.25  R: 9.2 – 14.1 | 11.64  SD = 1.47  R: 9.2 – 14.4 | p = 0.75 |
|  | HTC (%) | 32.9  SD = 3.6  R: 27.8 – 42.4 | 34  SD = 4.3  R: 27 - 41 | p = 0.90 |
| Day 4 | Hb (g/dl) | 10.66  SD = 1.46  R: 8.1 – 13.7 | 10.91  SD = 1.33  R: 8.3 – 13.6 | p = 0.92 (t-Stud) |
|  | HTC (%) | 31.8  SD = 4.16  R: 23.7 – 41.8 | 32  SD = 3.8  R: 24 - 40 | p = 0.79 |
| Discharge | Hb (g/dl) | 10.9  SD = 1.26  R: 9.1 – 13.7 | 11.16  SD = 1.14  R: 9.3 – 13.6 | p = 0.83 |
|  | HTC (%) | 32.5  SD = 3.44  R: 27.4 – 41.8 | 33  SD = 3.1  R: 28 - 40 | p = 0.78 |

**Suppl. Tab. 4a**

**Drainage output volume in milliliters (age 60-69 years old).**

| Postoperative day | Infiltrated group  (*n* = 13) | Not infiltrated group  (*n* = 12) | Statistics |
| --- | --- | --- | --- |
| Day 1 | 382  SD = 136  R: 110 - 580 | 431  SD = 127  R: 260 - 720 | p = 0.38 (t-Stud) |
| Day 2 | 156  SD = 76  R: 30 - 360 | 196  SD = 95  R: 80 - 450 | p = 0.27 (t-Stud) |
| Total | 538  SD = 161  R: 230 - 810 | 627  SD = 169  R: 340 - 940 | p = 0.21 (t-Stud) |

**Suppl. Tab. 4b**

**Drainage output volume in milliliters (age 70-79 years old).**

| Postoperative day | Infiltrated group  (*n* = 27) | Not infiltrated group  (*n* = 22) | Statistics |
| --- | --- | --- | --- |
| Day 1 | 356  SD = 121  R: 120 - 580 | 383  SD = 147  R: 70 - 670 | p = 0.5 (t-Stud) |
| Day 2 | 161  SD = 57  R: 70 - 300 | 179  SD = 62  R: 80 - 305 | p = 0.29 (t-Stud) |
| Total | 518  SD = 148  R: 190 - 800 | 562  SD = 142  R: 375 - 910 | p = 0.3 (t-Stud) |

**Suppl. Tab. 5a**

**Drainage output volume in milliliters corrected for acetabular size in millimeters (Ratio: drainage output volume/acetabular size) (age 60-69)**

| Postoperative day | Infiltrated group  (*n* = 11) | Not infiltrated group  (*n* = 10) | Statistics |
| --- | --- | --- | --- |
| Day 1 | 6.35  SD = 2.39  R: 1.06 – 10.17 | 7.76  SD = 2  R: 5.52 - 12 | p = 0.19 |
| Day 2 | 2.75  SD = 1.37  R: 0.53 - 6 | 3.67  SD = 1.78  R: 1.53 – 8.33 | p = 0.79 |
| Total | 9.11  SD = 2.83  R: 4.1 – 14.46 | 11.44  SD = 2.67  R: 8.27 – 15.74 | p = 0.29 |

**Suppl. Tab. 5b**

**Drainage output volume in milliliters corrected for acetabular size in millimeters (Ratio: drainage output volume/acetabular size) (age 69-79)**

| Postoperative day | Infiltrated group  (*n* = 18) | Not infiltrated group  (*n* = 18) | Statistics |
| --- | --- | --- | --- |
| Day 1 | 6.73  SD = 2.05  R: 4.03 – 10.64 | 6.9  SD = 2.2  R: 1.89 – 11.16 | p = 0.93 |
| Day 2 | 3.06  SD = 1.1  R: 1.42 – 5.77 | 3.13  SD = 1.14  R: 1.66 – 5.6 | p = 0.97 |
| Total | 9.8  SD = 2.57  R: 6.35 – 15.38 | 10.04  SD = 2.13  R: 6.55 – 15.16 | p = 0.71 |

**Suppl. Tab. 6a**

**Comparison of estimated blood loss in milliliters between groups (age 60-69)**

|  | Infiltrated group  (*n* = 13) | Not infiltrated group  (*n* = 12) | Statistics |
| --- | --- | --- | --- |
| Day 1 | 699.9  SD = 147.73  R: 477.6 – 962.1 | 576.35  SD = 255.59  R: 154.1 – 1037.9 | p = 0.86 |
| Total | 1288.62  SD = 489.5  R: 662.5 – 2353.81 | 1288.96  SD = 427.94  R: 682.94 – 1995.83 | p = 0.96 |

**Suppl. Tab. 6b**

**Comparison of estimated blood loss in milliliters between groups (age 70-79)**

|  | Infiltrated group  (*n* = 27) | Not infiltrated group  (*n* = 12) | Statistics |
| --- | --- | --- | --- |
| Day 1 | 512.9  SD = 275.17  R: 104.88 – 1232.3 | 461.1  SD = 295.07  R: 67.22 – 1138.24 | p = 0.86 |
| Total | 1157.13  SD = 456.95  R: 525.1 – 2410.23 | 1174.4  SD = 373  R: 544.1 – 2006.17 | p = 0.26 |

**Suppl. Tab. 7**

**Estimated total blood loss in milliliters corrected for acetabular size in millimeters (Ratio: blood loss/acetabular size) (age 69-79)**

| Age group | Infiltrated group | Not infiltrated group | Statistics |
| --- | --- | --- | --- |
| 60-67 years | 20.54  SD = 7.26  R: 7.59 – 32.1 | 21.5  SD = 6.53  R: 11.77 – 28.9 | p = 0.99 |
| 70-79 years | 20.79  SD = 7.66  R: 10.1 – 39.16 | 21.72  SD = 6.31  R: 12.36 – 34.59 | p = 0.58 |
