# Supplementary material for: Local infiltration analgesia with bupivacaine and adrenaline does not reduce perioperative blood loss in total hip arthroplasty
Source: PLoS One. 2021 Sep 9;16(9):e0257202. doi: 10.1371/journal.pone.0257202 (PMC8428560; doi:10.1371/journal.pone.0257202)
Supplement: Attachment — Submitted filename: Response to the reviewers.docx [file pone.0257202.s003.docx]

The authors wish to thank for a kind and meaningful review. We did our best to fulfill all the requirements and address comments properly. All the reviewer comments were taken into account.

**Journal Requirements:**

Please provide additional details regarding participant consent.

Patients provided an informed written consent to allow their medical data to be analyzed in this study. This study was approved by the local Bioethics Committee (issued 25.11.2009, NKEBN/332/2009). – This statement is included now in the Material and Methods section. I am sorry to have not included it previously as it should have been.

**Reviewer #1:** General comments
As long as wound infiltration with local anesthetics is no longer necessary for early postoperative pain control after development of local nerve blocks (lines 70-72),\n what is the importance of the current research?

Thank you for this comment. The idea behind this study was whether LIA with adrenaline would provide additional benefit compared to local nerve blocks by limiting perioperative blood loss and justify returning to LIA with adrenaline, disregarding the local nerve blocks. This is stated clearly in the introduction section under the hypothesis.

*Abstract*
. Line 23: meaning of the abbreviations Hb, HTC or RBC \nwere not previously reported - corrected
. Lines 24-25: “amount of blood transfusions on the 1st, 4th or at patients’ discharge between infiltrated and not infiltrated groups”. \nshould be corrected to” the 1st or 4th post-operative days” – corrected
*Introduction*
. Introduction must highlight the importance of the current research. The authors in lines 42-44: In total hip arthroplasty, local infiltration analgesia with adrenaline has proven to be \nbeneficial in postoperative pain control [7,12], but it failed to show a consistent effect on bleeding [3,13,14]. \nSo, what is the clinical significance of the current research? – other studies used different solutions and application protocols as in this study

*Material and Methods*
The 2 groups are not operated by the same surgeon and this is one of the limitations of the study, it is not a randomization and not a point of strength - this expression has been removed; the discussion section explaining this matter has been updated to discuss this limitation.

Line 99: Meaning of abbreviation “BMI” was not previously mentioned. – corrected for “body mass index (BMI)”

*Results*
. Line 137: “There no significant differences in the female to male ratio in both groups (2x2 chi-square test “\n
. Authors did not report use of Chi – square test in Statistical methods (lines 130-133) – the use of this test is mentioned in this section
. Line 169: “Ratio = drainage output volume (ml)/acetabular size (mm)” Was this equation developed by the authors? \n – the formula was developed by the authors, this fact is mentioned in the text now

*Discussion*
Is too long and should be shortened – the discussion was updated and redundant information excluded.
. Line 226: “The study is not a randomized controlled study, but some criteria of randomization are met”. This sentence is not true and must be deleted. \n – this section in the discussion has been updated.

*Tables*
Considering each table as a separate entity, meaning of all abbreviations used in each table must be reported in a footnote after each table. – the footnotes were applied where appropriate

**Reviewer #2**: The authors evaluated whether local infiltration analgesia with bupivacaine and adrenaline reduced perioperative blood loss in THA. They compared a group with the analgesia and the other without it in a retrospective manner and found that there were no significant differences between the two groups. This is an interesting study which could answer this clinical question in a straightforward manner. However, this manuscript has obvious limitations and issues to be solved.

1, To answer a certain clinical question by a retrospective analysis, one needs to have a clear hypothesis BEFORE the start of the study. What was it in this study? Please describe it clearly.

The hypothesis is described at the end of the introduction.

2, Then, the researcher(s) must calculate the sample size depending on the hypothesis according to previous similar reports. Please describe the calculation in this study.

The sample size was calculated according to previous reports and was considered adequately powered.

3, The blood loss should be estimated not only the drainage output but also calculated by the differences between preoperative and postoperative Hb, HTC, and RBC. Moreover, the estimated blood loss can be calculated by established formulae using those values. Please show these results.

The comparison of preoperative and postoperative values of Hb, HTC and RBC are provided in tables 2 and 3. The blood loss was estimated according to an appropriate formula by Meunier et al. (source cited in the article) and compared between groups in an additional table (Tab. 6).
