# Supplementary material for: Local infiltration analgesia with bupivacaine and adrenaline does not reduce perioperative blood loss in total hip arthroplasty
Source: PLoS One. 2021 Sep 9;16(9):e0257202. doi: 10.1371/journal.pone.0257202 (PMC8428560; doi:10.1371/journal.pone.0257202)
Supplement: Attachment — Submitted filename: Response to the reviewers 2.docx [file pone.0257202.s004.docx]

The authors wish to thank for a kind and meaningful review. We did our best to fulfill all the requirements and address comments properly. All the reviewer comments were taken into account.

Reviewer #3: • I see the senior surgeon of both groups is different. Are the bleeding control practices of both surgeons similar during the patients' operations? For example; In the hospital where I work for total hip replacement surgery, there are two kind of surgeons one does this surgery in half an hour and the other one in 2 hours. The approaches of these two surgeons to bleeding and acetabular reaming are completely different. For these and similar reasons, it would not be correct to compare the patients of these two surgeons in the same study. In my opinion, the fact that two different surgeons have two different applications does not mean that it is appropriate to compare these two different groups. As a result, this situation causes bias in the study. This is huge one of the limitations of the present study.

We agree that the fact that both groups were not operated by a single surgeon is a limitation of this study, but this limitation is difficult to overcome in a retrospective analysis such as this study. The senior surgeons, as well as the whole operating team, has similar bleeding control practices, as they learned their craft and performed surgeries in the same center all their professional careers. The surgeries were performed by senior residents, who learned their basic skills, such as surgical approaches and bleeding control, from both senior supervising surgeons (KK, MT). The senior residents operated patients in both groups, therefore the operating surgeon bias is partially avoided. None of the patients in this study were operated on the supervising surgeons personally; they were the first surgical assistants and their role was to guide the residents with their experience during surgeries. The senior residents (all of them in their 4^th^ or 5^th^ year of residency with several hundred different surgeries performed personally) already have their bleeding practices well established, therefore the supervising surgeons’ influence on their basic performance, including bleeding control practices, is very limited. Therefore we believe that the influence of the surgeons’ experience and bleeding control practices have very little if any influence on the results of the study.

Additionally, we compared surgical time in both groups and there were no significant differences between them. This may be considered an indirect indication of similar bleeding control practices, as significant differences in this area would result in longer operating times when a more meticulous and time consuming bleeding control was done in one of the groups.

• Another limitation of the study is the significant difference in acetabular sizes between the two groups.

This is probably the most difficult limitation to overcome in this study. We did our best to at least partially overcome this problem by correcting the total drainage output by acetabular size as seen in tab. 5. Moreover, in the revised manuscript we performed a similar correction of total estimated blood loss (the most important outcome criterion in our opinion) by acetabular size to further check its influence on the results. No significant differences were found in this regard, as seen at the end of the Results section (below Tab. 6). A short comment was added in the discussion section.

• Line 215: " ... randomized controlled study by Villatte et al. ... " The study about what? – an explanation is provided in the appropriate location

Reviewer #4: Dear Authors
I congratulate you for your valuable study examining the effect of bupivacaine and adrenaline applied during total hip arthroplasty surgery on postoperative blood loss. I think some methodological adjustments are needed for your study to be published. My recommendations are as follows:

1. Keywords are not suitable for MESH. I recommend using “<https://meshb.nlm.nih.gov/search>” for keyword selection to increase the scannability of the article.

The keywords were updated and checked in MESH. The terms: total hip arthroplasty, bleeding, hemostasis, local anesthesia, can be found in MESH.

2. Comorbidity was not included in the exclusion criteria. An explanatory sentence might be appropriate in this regard.

A short explanation of patients’ preparation for surgery with regard to their general health status was added to the Materials and Methods section. Patients with general contraindications for surgery were not operated on and therefore not included in the study.

3. The age range was kept very wide in the selection of the patients included in the study. Such a wide age range may make a difference in terms of susceptibility to coagulopathy. Analysis by age groups can improve the quality of the study.

An analysis in two age groups, that is between 60 and 69 years of age and 70-79 years of age, was performed separately. The number of patients above and below this age threshold was too limited for evaluation. A short remark about those results was added in the results as well as in the discussion section. Generally, the results were similar as in the general evaluation. To avoid too much specific data, the results in tables were added as supplementary material.

4. Is there a difference between the two groups in terms of surgical time? No information was given about this. Extended surgical time is a factor that increases bleeding. – The operative time was included in patient comparison, no significant differences were found between groups.

5. Is the content of the injected solution (The local anesthetic mixture was prepared with 100 ml saline solution with 50 mg of bupivacaine and 1 mg adrenaline) a standard protocol defined in the literature or is it the surgeon's preference?

This solution was prepared according to a standard developed in our institution, and is similar to the solutions used in other studies – this sentence is now included in the Material and Methods section.

6. “In the infiltrated group 8 patients and in the not infiltrated group 5 patients had their stay prolonged above Day 4.” The sentence should be in the results section. – after careful consideration we removed this sentence completely, as this information is irrelevant to the results of the study and is not discussed at any point in the manuscript.

7. BMI and blood loss can be compared. The correction calculation made for the acetabular size can also be made for the BMI.

In this study, the acetabular size was the only significant difference between groups in the patients’ characteristics. There were no differences in age, weight, BMI and stem size, all of which can theoretically influence postoperative bleeding.

The the idea behind the correction for acetabular size was to minimize the influence of the size differences between groups on the results. There were no significant differences between BMI results between groups, therefore we believe that a direct comparison of the results was enough and no correction for the BMI results was needed.

We performed the correction as requested, but taking into account all the above mentioned arguments, decided not to include it in the main body of the manuscript. It does not change the results of the study and the conclusion in any way, and the inclusion of this information would make the already complicated manuscript even more difficult to read. The calculation is done in the Suppl. 1 data file, including statistical comparison.

However, since the acetabular sizes may be of great importance in the results, we corrected the total estimated blood loss for acetabular size and included the results in the manuscript. This is the most important parameter in this study and we believe that this additional information may increase the value of the results.
